# The Molecular and Mechanistic Insights Based on Gut–Liver Axis: Nutritional Target for Non-Alcoholic Fatty Liver Disease (NAFLD) Improvement

**DOI:** 10.3390/ijms21093066

**Published:** 2020-04-26

**Authors:** Yun Ji, Yue Yin, Lijun Sun, Weizhen Zhang

**Affiliations:** Key Laboratory of Molecular Cardiovascular Science, Ministry of Education, Department of Physiology and Pathophysiology, Peking University Health Science Center, Beijing 100191, China; jean500@163.com (Y.J.); sunlj9002@163.com (L.S.)

**Keywords:** gut microbiota, gut–liver axis, liver disease, nutrition, barrier function

## Abstract

Non-alcoholic fatty liver disease (NAFLD) is recognized as the most frequent classification of liver disease around the globe. Along with the sequencing technologies, gut microbiota has been regarded as a vital factor for the maintenance of human and animal health and the mediation of multiple diseases. The modulation of gut microbiota as a mechanism affecting the pathogenesis of NAFLD is becoming a growing area of concern. Recent advances in the communication between gut and hepatic tissue pave novel ways to better explain the molecular mechanisms regarding the pathological physiology of NAFLD. In this review, we recapitulate the current knowledge of the mechanisms correlated with the development and progression of NAFLD regulated by the gut microbiome and gut–liver axis, which may provide crucial therapeutic strategies for NAFLD. These mechanisms predominantly involve: (1) the alteration in gut microbiome profile; (2) the effects of components and metabolites from gut bacteria (e.g., lipopolysaccharides (LPS), trimethylamine-N-oxide (TMAO), and N,N,N-trimethyl-5-aminovaleric acid (TMAVA)); and (3) the impairment of intestinal barrier function and bile acid homeostasis. In particular, the prevention and therapy of NAFLD assisted by nutritional strategies are highlighted, including probiotics, functional oligosaccharides, dietary fibers, ω-3 polyunsaturated fatty acids, functional amino acids (L-tryptophan and L-glutamine), carotenoids, and polyphenols, based on the targets excavated from the gut–liver axis.

## 1. Introduction

Ranging from hepatic steatosis and steatohepatitis to fibrosis, non-alcoholic fatty liver disease (NAFLD) is classified as a metabolic dysfunction-associated liver disease, where fat accumulation in hepatocytes exceeds 5% of the weight of liver in the absence of excessive alcohol consumption and other stimulating factors (e.g., drugs and virus) [1]. The global prevalence of NAFLD among human beings over 15 years of age is estimated to be 33.5% in 2030 [2]. The pathological mechanism concerning the onset and progression of NAFLD is complicated and has not been fully elucidated. However, a multiple-hit hypothesis has been accepted to explain the key factors involved in the metabolic and molecular mechanism of NAFLD. These factors mainly comprise genetic and epigenetic factors, dietary determinants, insulin resistance, lipotoxicity, pro-inflammatory factors, and gut microbiome [3]. Regarding the innate risk factors, the candidate genes screened by genome wide association studies (GWAS) have enabled the correlation of genetic variants with NAFLD. For example, variants in *PNPLA3* (Patatin-like phospholipase domain-containing 3) and *TM6SF2* (transmembrane 6 superfamily member 2) have been tightly associated with the occurrence and progression of NAFLD [4,5]. Moreover, the inherited phenomenon of epigenetics regulating gene expression through DNA methylation, histone modifications, and microRNAs without changing the DNA sequence also offers an insight on NAFLD pathogenesis [6]. Dietary pattern is a key precipitating factor for NAFLD. Long-term excessive intake of calories especially diet high in refined carbohydrates, saturated fat, or fructose has been highly linked to NAFLD. This dietary pattern may result in insulin resistance and a disorder in lipid metabolism, leading to hepatic steatosis, inflammation, and fibrosis. Of particular note, prolonged consumption of excess calories may elicit an imbalance in gut microbiota [7,8,9,10], which has a profound effect on the progression of NAFLD.

The intestinal tract of humans and animals is colonized by a diverse range of microbes. These microbes play a critical role in maintenance of gut function, modulation of host immune response and chronic diseases (e.g., obesity, diabetes, and fatty liver), as well as metabolic homeostasis [11,12,13]. The changing of the composition of gut microbiota has been accepted as an effective therapy to regulate the development of obesity [14,15]. Generally, NAFLD appears as a widespread and evident adverse effect of obesity [16,17]. Emerging human and rodent studies on obesity-related metabolic diseases have revealed the pivotal role of gut microbiome in NAFLD pathogenesis. [18,19,20]. The liver of patients with NAFLD are frequently affected by the detrimental effects from the altered gut microbiome through the gut–liver axis. Together with intestinal dysbacteria, NAFLD patients present impaired intestinal barrier function and bile acid homeostasis as well as increased translocation of bacteria and bacteria-derived products into the liver. Dietary supplements, such as probiotics, functional oligosaccharides, dietary fibers, ω-3 polyunsaturated fatty acids, functional amino acids (L-tryptophan and L-glutamine), carotenoids, and polyphenols, gradually help to normalize the gut microbiome and reinforce the intestinal barrier function and bile acid homeostasis, which provide the potential to reduce the unwholesome products from gut microbiota [21,22,23]. Expanding the knowledge of interactions between nutraceuticals and the gut–liver axis, followed by the development of specific nutritional interventions, will boost the health status of NAFLD individuals.

## 2. Gut–Liver Axis Is the Bridge between Gut Microbes and Liver

Over the last decade, the term “gut–liver axis” has emerged from a collection of evidence that expanding the influence of gut microbiota-generated components and metabolites, intestinal barrier permeability, and bacterial translocation on liver diseases. Venous blood carries nutrients absorbed from food, factors derived from intestinal microbiota, and immune response products, which enters the hepatic tissue through the portal vein [24]. Meanwhile, bile acids synthesized in hepatocytes are conjugated with glycine or taurine, forming bile salts in the liver and being stored in gallbladder and then passing into the small intestine [25]. This close connection between gut and liver determines the crucial regulatory effect of the gut microbiota on liver health. Long-term consumption of diet high in calories and saturated fat may lead to dysbiosis in gut microbiota; this in turn evokes imbalanced bile acid pool and intestinal barrier dysfunction, followed by an increase in bacterial translocation and pro-inflammatory components and metabolites from bacteria entering into the liver. Ultimately, it may induce an acceleration in occurrence and development of NAFLD.

### 2.1. Gut Microbiome Profiles in NAFLD Patients

*Bacteroidetes* and *Firmicutes* account for the majority (above 90%) of human gut microbiota, while other phylums of bacteria exist in small amount, including *Proteobacteria*, *Verrucomicrobia*, *Actinobacteria*, *Fusobacteria* and *Cyanobacteria* [26]. Three major classes (*Bacilli*, *Clostridia*, and *Mollicutes*) encompassing over 250 genera are included in *Firmicutes*, almost all of which are gram-positive [27]. In contrast, all gut bacteria belonging to the phylum of *Bacteroidetes* are gram-negative, composed mainly of the genera of *Bacteroides*, *Prevotella*, *Alistipes*, and *Parabacteroides* [28]. Accumulating clinical and animal studies have indicated that NAFLD is intimately associated with disruption of the balance between *Firmicutes* and *Bacteroidetes*. Although available studies have pointed out the association between the increased abundance of gut *Firmicutes* and NAFLD, other findings confirm that overgrowth of *Bacteroides* plays a key role in the development of NAFLD [29,30,31,32]. In addition, the severity of NAFLD is associated with an increase in fecal abundance of *Bacteroides*, accompanied by a decrease in the level of *Prevotella* [33]. Other evidence suggests the fecal abundance of *Anaerosporobacter* and *Faecalibacterium* are lower, whereas *Allisonella* and *Parabacteroides* are higher in non-alcoholic steatohepatitis (NASH) patients [31]. Metagenome sequencing revealed *Bacteroides vulgatus* and *Eubacterium rectale* present the highest abundance in the feces from mild and moderate NAFLD, while *Bacteroides vulgatus* and *Escherichia coli* reach the most abundant in liver fibrosis [34]. To sum up, the above evidence supports the theory that the development and progression of NAFLD is closely linked to gut microbiota dysbiosis.

### 2.2. Gut Microbiota-Derived Components and Metabolites That Accelerating NAFLD

A disturbance of the intestinal microbiota in response to unbalanced diet (e.g., diet high in saturated fat and fructose) may elicit an increase in intestinal permeability, leading to chronic inflammatory condition [35]. Remarkably, a continuous low-grade inflammatory state may result in an acceleration in the progression from hepatic simple steatosis to NASH [36,37]. Serving as a key constituent of the outer membrane of cell wall in most gram-negative bacteria, lipopolysaccharides (LPS) (also known as endotoxins) has well been identified as a major factor in activation of innate immunity and are therefore suggested as a vital pathogenesis for NAFLD [38,39]. Indeed, patients with hepatic steatosis and inflammation present an elevation in the serum LPS [40]. This may be associated with a rise in the abundance of specific gram-negative bacterial genus, including *Bacteroides*, *Enterobacteria*, *Escherichia*, and *Proteus*, as observed in NAFLD patients [33,41]. Recently, study on germfree mice have uncovered that colonization of one of the three nonvirulent LPS-producing strains identified from obese human gut induces NAFLD in combination with high-fat diet feeding [42]. These results were further corroborated by either interference in the synthetic pathway of bacterial LPS or deletion of the LPS receptor toll-like receptor 4 (TLR4). As a main TLR4 ligand governing the release of pro-inflammatory cytokines, LPS can potentiate the susceptibility of NAFLD through the augmentation of inflammatory responses [43,44]. In addition to LPS, other pro-inflammatory bacterial components such as peptidoglycans, lipoteichoic acids, bacterial DNA, and extracellular vesicles have also been noted in recent years [45]. However, the underlying mechanism by which the role of these components produced by specific gut bacterial strains in NAFLD requires more extensive and explicit studies.

Considerable attention has been also paid to the role of gut microbiota-derived metabolites in the pathological process of NAFLD. A clinical research with 330 subjects by Barrea et al. reveals that the circulating levels of trimethylamine-N-oxide (TMAO) is a novel indicator of metabolic syndrome and NAFLD [46]. Although TMAO is synthesized in the liver, trimethylamine (TMA), the precursor of TMAO, is generated from gut bacteria. L-carnitine, choline, or betaine have been the major substrates for TMA synthesis by gut bacterial strains (e.g., *Clostridium asparagiforme*, *Clostridium sporogenes*, *Clostridium hathewayi*, *Escherichia fergusonii*, *Anaerococcus hydrogenalis*, and *Proteus penneri*) [47]. TMAO is formed by the oxidation of TMA following the catalysis of flavin-containing monooxygenase (FMO) enzymes in the liver [48]. The precise mechanisms involving the relationship between TMAO and the initiation and progression of NAFLD remains to be clarified. However, it has been verified that TMAO exacerbates hepatic steatosis by blocking the farnesoid X receptor (FXR) signaling activated by bile acid [49]. By contrast, the activation of FXR signaling have shown a protective effect against NAFLD including liver steatosis and inflammation [50,51]. It should be mentioned that N,N,N-trimethyl-5-aminovaleric acid (TMAVA), a newly identified gut microbiota metabolite, appears as a key molecule exacerbating high-fat diet-induced liver steatosis through the inhibition of carnitine synthesis accompanied by a reduction in mitochondrial fatty acid β-oxidation in hepatic tissue [52]. This metabolite is yielded by *Enterococcus faecalis* and *Pseudomonas aeruginosa* using trimethyllysine [52]. In future study, more metabolites are required to be identified and examined from particular gut strains to provide more extensive knowledge concerning the interaction of gut microbes with NAFLD.

### 2.3. Impaired Intestinal Barrier Function is an Important Reason for the Development of NAFLD

Intestinal barrier performs an effective defensive action against the translocation of harmful substances including bacteria, bowel luminal antigens and inflammatory factors, and is usually assessed by intestinal permeability [53]. Gut microflora dysbiosis has been closely followed by increased intestinal permeability and subsequent bacterial translocation to the liver, leading to the release of inflammatory cytokines and free radicals from activated Kupffer cells [54]. Ample evidence reveals that NAFLD individuals develop an imbalanced gut microbiome and compromised intestinal permeability [55,56,57]. Inflammatory bowel disease (IBD) patients have a high incidence (up to 33.6%) of NAFLD even independent of metabolic risk [58], which has been closely associated with impaired intestinal barrier function. An impairment of the integrity of intestinal barrier initiated by disordered gut microbiome has been a prerequisite for NASH [59]. Consequently, interventions preserving the integrity of the intestinal barrier may contribute to attenuating or preventing the progression of NAFLD. It should be noted that zonulin serves as a key protein regulating intestinal permeability reversibly by adjusting the size of tight junctions between epithelial cells [60,61]. Serum circulation levels of zonulin present a significant positive correlation with pathological indicators in NAFLD, especially in NASH [62]. Of note, it has been shown that the endogenous ethanol content of NAFLD patients is substantially higher than that of healthy individuals [63]. The impaired intestinal integrity is partially associated with the endogenous production of ethanol. Intriguingly, recent evidence found that specific gut strains (*Klebsiella pneumoniae*) from NAFLD patients possess the ability to produce high level of endogenous ethanol, thus exacerbating the compromised intestinal barrier function and hepatic steatosis [64]. 

### 2.4. Gut–Liver Crosstalk Mediated by Bile Acids

The circulation of bile acids between liver and intestine is quite active. Bile acids are known as amphipathic hydroxylated steroids synthesized from catabolism of cholesterol in the liver and are released into the small intestine in the form of bile salts from gallbladder [65]. They function not only to facilitate the emulsification, transport, and absorption of lipids and fat-soluble vitamins but also to regulate the balance of glucolipid metabolism and immune responses [66]. The synthesis of primary bile acids cholic acid (CA) from cholesterol is initiated by rate-limiting enzyme cholesterol 7a-hydroxylase (CYP7A1) in hepatocytes [67]. Alternatively, sterol-27-hydroxylase (CYP27A1) catalyzes the production of chenodeoxycholic acid (CDCA) or muricholic acids (MCAs, only in mice) from cholesterol [68]. The formation of secondary bile acids (deoxycholic acid (DCA), lithocholic acid (LCA), and ursodeoxycholic acid (UDCA)) is achieved via the modification of bile salts (primary bile acids conjugated with glycine or taurine) by gut microbiota [69]. In mice, other secondary bile acids including Omega-MCA (ωMCA), hyocholic acid (HCA), murideoxycholic acid (MDCA), and hyodeoxycholic acid (HDCA) are formed from MCAs by microbial epimerization and dehydroxylation [68]. About 95% of the bile acids are reabsorbed into the terminal ileum and are transferred to the liver via the portal vein [70]. Importantly, bile acids regulate bile acid receptors including FXR and Takeda G-protein receptor 5 (TGR5) which are known to modulate insulin sensitivity, glucolipid metabolism, energy homoeostasis, inflammatory responses, and intestinal barrier function [71,72,73,74]. FXR and TGR5, in turn, play a crucial role in the feedback regulation of bile acid homeostasis [75]. Imbalance of intestinal flora may lead to abnormal size and composition of bile acids and dysregulation of FXR and TGR5 signaling [76]. Specific bile acid conjugates esteem differential feedback of regulatory influences in FXR signaling. Since the FXR is a primary controlling actor for bile acid synthesis and bile flow, this would imply that patients with NAFLD manifest altered bile acid kinetics. For instance, a metabolomic study revealed that NASH patients presented higher plasma concentration of glycine or taurine-conjugated CA, and glycine-conjugated CDCA, compared with healthy subjects [77]. FXR and TGR5 have become important intervention targets for NAFLD [78]. Activation of FXR ameliorates NASH through multiple mechanisms, including the suppression of monocyte and neutrophil infiltration and the attenuation of NF-κB-mediated inflammation signaling [50]. TGR5 activation by agonist also has been proved to improve liver steatosis in a mouse model of NAFLD [79]. 

## 3. Targeting the Gut–Liver Axis as a Nutritional Treatment for NAFLD

Disturbances in intestinal flora composition and gut–liver axis following unbalanced diets has a profound influence on the progression of NAFLD. However, a large number of dietary supplements supporting the homeostasis of intestinal bacteria have been reported, including probiotics, functional oligosaccharides, dietary fibers, ω-3 polyunsaturated fatty acids (ω-3 PUFAs), functional amino acids (L-tryptophan and L-glutamine), carotenoids, and polyphenols. These nutritional interventions have been turned out to protect and improve NAFLD through targeting the gut–liver axis, including (1) balance of gut microbiome; (2) preservation of bile acid homeostasis; (3) reinforcement of intestinal barrier function; (4) reduction in bacterial translocation; (5) decrease in the unwholesome components and metabolites from gut bacteria; and (6) supply of gut bacteria-derived beneficial metabolites (e.g., short chain fatty acids (SCFAs), indoles, and urolithins).

### 3.1. Probiotics

The use of probiotics has become an enticing and promising approach for the prevention and therapy of NAFLD. Probiotics refer to the live microorganism strains with sufficient quantity which provide the host with a health benefit or improved pathological conditions [80]. These strains induce a competitive exclusion of pathogenic bacteria to ensure a healthy and balanced intestinal microflora ecosystem favoring epithelial barrier and host immune function [81,82]. Plentiful studies have shown the extensive salubrious effects of probiotics (e.g., *Lactobacillus* and *Bifidobacterium*) on liver disease. As shown in high-fructose or high-fat diet-induced experimental NAFLD models, *Lactobacillus rhamnosus GG* ameliorated NAFLD by (1) control of gut microbiome; (2) repair of intestinal barrier; and (3) suppression of hepatic steatosis, inflammation, and lipid accumulation [83,84]. *Bifidobacterium* serves to protect against secretion of pro-inflammatory cytokines and dysfunction in intestinal barrier both in vitro and in vivo [85]. Results from a randomized clinical trial indicates that patients with NAFLD receiving a complex-probiotic prepared by 14 probiotic bacteria genera belonging to *Lactobacillus*, *Lactococcus*, *Bifidobacterium*, and *Propionibacterium* presented a reduction in fat accumulation in liver and aminotransferase activity, and pro-inflammatory factor levels including tumor necrosis factor-α (TNF-α) and interleukin 6 (IL-6) in serum [86]. However, probiotics therapy to NAFLD probably is not involved in the regulation of TMAO levels, according to the observations from high-fat diet feeding mice and patients with metabolic syndrome [87,88]. As for bile acids metabolism, a randomized double-blind crossover trial revealed that consumption of *Bacillus subtilis R0179* and *Bifidobacterium animalis subsp. lactis. B94* for 6 weeks showed a significant increase in serum deconjugated bile acids [89]. Evidence from mice indicated that administration of a probiotic mixture of VSL#3 facilitated hepatic synthesis and fecal excretion of bile acids via down-regulation of fibroblast growth factor 15 (Fgf15) [90], while Fgf15 is known to expedite hepatic fibrosis and even cancer in the background of NAFLD [91,92].

### 3.2. Dietary Fibers

Dietary fiber is characterized as nondigestible carbohydrate polymers containing three or more monomeric units that are included in plant diets such as grains, legumes, fruit, and vegetables [93,94]. Comparative nutritional evaluation studies suggested that patients with NAFLD consumed less dietary fiber than healthy individuals [95,96]. In comparison, the consumption of dietary fiber has been a favorable factor resistant to the progression of NAFLD [97]. Fibers leaving the upper gastrointestinal tract are degraded into SCFAs including acetate, butyrate, and propionate through the fermentation of gut microbiota at the lower part of the gastrointestinal tract [98]. Such SCFAs participate in a broad variety of physiological processes related to metabolism, immunity, endocrine, and inflammation [99]. Furthermore, dietary fibers serve to adsorb bile acids and cholesterol in the gastrointestinal tract, thus changing the bioavailability of secondary metabolites and playing a role in management of hyperlipidemia [100,101]. An improvement in fatty livers index, hepatic steatosis index, and NAFLD hepatic fat score have been shown by subjects who consume higher insoluble fiber (≥7.5 g/day) [102]. Consumption of insoluble dietary fiber from soy hull induced an alteration in fecal microbiota composition, in particular the abundance of *Lactobacillales* and *Bifidobacteriales* known as probiotics [103]. Additionally, the increase in nutritional fibers improve hepatic steatosis and liver function affiliated with dramatically lowered serum level of zonulin protein, denoting an enhanced barrier function and thus a reduced intestinal permeability [104].

### 3.3. Functional Oligosaccharides

Functional oligosaccharides have become an increasing focus of interest in NAFLD amelioration. Functional oligosaccharides (e.g., fructo-oligosaccharides, galacto-oligosaccharides and chitosan oligosaccharides) are prebiotics that have been shown (1) to facilitate the proliferation of beneficial flora in gut; (2) to suppress pathogenic bacteria; and (3) to participate in the control of glucolipid metabolism, immunity and oxidative injury [105,106]. Dietary fructo-oligosaccharides contribute to the recovery of normal intestinal microbiome and intestinal barrier function, thereby alleviating steatohepatitis in a methionine-choline-deficient mouse model [107]. Moreover, a fructo-oligosaccharides supply reversed high-fat diet feeding-induced reduction in level of *Bifidobacteria* and increase in plasma endotoxin content in mice [108]. Evidence from integrated lipidome and gut microbiome uncovered that chitosan oligosaccharide serves to ameliorate hepatic injury, steatosis, and inflammation as well as to reinforce the intestinal barrier [109]. Also, this oligosaccharide induced a reduction in the cecal abundance of *Mucispirillum* and an elevation in the content of *Coprococcus* [109]. Besides, the contribution of oligosaccharides to bile acid secretion and bile acid pool kinetics has been demonstrated in various animal models [110,111,112]. Overall, the findings from above animal studies hint that inclusion of oligosaccharides in food is a healthy way of providing beneficial effects in liver health. Future study is expected to evaluate the efficacy of oligosaccharides in NAFLD patients through clinical trials.

### 3.4. Functional Amino Acids (l-tryptophan and l-glutamine)

L-tryptophan is an amino acid essential for human and animals and exists in foods derived from protein such as meats, milk, nuts, and seeds [113]. The biochemical and physiological functions of tryptophan are not only restricted to its host metabolites (kynurenine, serotonin, and melatonin), but a wide variety of gut microbiota-sourced metabolites have also been identified [114,115]. Through the catalysis of enzymes expressed in gut microbiota, dietary tryptophan is converted into indole and its derivatives (indole lactic acid (ILA), indole acetic acid (IAA), indole aldehyde (IAld), indole propionic acid (IPA), and indoleacrylic acid (IA)). Currently, various evidence has associated indoles with the improvement of gut and liver health. For instance, IAld induced STAT3 (signal transducer and activator of transcription 3) phosphorylation by stimulating the secretion of interleukin-22 (IL-22) via aryl hydrocarbon receptor (AhR) to promote the proliferation of the intestinal epithelial cells, which in turn restores the barrier function [116]. Treatment with indole attenuated high-fat diet-induced severity of hepatic steatosis and inflammation in mice, which was connected with the regulation of PFKFB3 (6-Phosphofructo-2-Kinase/Fructose-2,6-Biphosphatase 3) and the normalization of gut microbiome [117]. Study on rats revealed that IPA facilitated the protection against steatohepatitis induced by high-fat diet, as demonstrated by the improvement of gut dysbiosis and intestinal epithelial barrier function and the inhibition of pro-inflammatory signaling [118]. In addition, it has been shown that IAA displays anti-oxidative and anti-inflammatory activity, which is beneficial in the resistance to NAFLD induced by high-fat feeding and pro-inflammatory response in macrophages [119,120]. Interestingly, *Bifidobacterium* strains collected from human infants have been turned out to produce ILA [121], the favorable effect of these strains may be associated with ILA, which deserves to be corroborated in future studies. L-glutamine is the most plentiful amino acid in the body dispersed predominantly in the skeletal muscle, small intestine, liver, and kidney [122]. This amino acid serves as a substrate for the synthesis of antioxidant glutathione [123]. Importantly, supplementation with parenteral glutamine improved intestinal barrier function and decreased the occurrence of infection [124]. These effects of glutamine are not only mediated by the redox status, but also depend on its regulation on immune response and signaling pathways (e.g., PI3K (phosphatidylinositol 3-kinase)/PKB (protein kinase B)/mTOR (mechanistic target of rapamycin)) [122,125]. Obese adults receiving l-glutamine for 14 consecutive day exhibited a reduction in fecal abundance of *Dialister*, *Dorea*, *Pseudobutyrivibrio*, and *Veillonella*, and an increase in *Prevotella*, compared with the overweight individuals daily supplemented with equivalent l-alanine [126], suggesting a modulation on the composition of gut microbiota by l-glutamine. Although more clinical and mechanistic studies in relation to the effect of l-glutamine on NAFLD remain to be performed, research on mice has shown a protective role of oral administration of L-glutamine against western-style diet-induced NASH [127].

### 3.5. Omega-3 Polyunsaturated Fatty Acids

The source of ω-3 PUFAs include alpha-linolenic acid (ALA, 18:3) in plant oils and eicosapentaenoic acid (EPA, 20:5) and docosahexaenoic acid (DHA, 22:6) in fish oils. In contrast to the pro-inflammatory effect of ω-6 PUFAs (linoleic acid (18:2), γ-linolenic acid (18:3), arachidonic acid (20:4)), ω-3 PUFA (especially EPA and DHA) are well known to mitigate inflammation [128]. As a result, an increase in dietary intake ratio of ω-6/ω-3 PUFAs has been associated with various pathogenesis of diseases such as cardiovascular disease, arthritis, obesity, diabetes, and liver disease [129,130,131,132,133]. ω-3 PUFAs hold the potential to be a supportive option for the prevention and therapy of NAFLD as observed in various clinical and animal studies [134,135,136,137]. Previous studies have shown an improvement effect of ω-3 PUFAs on hepatic enzymes and triglycerides in liver and serum [138]. In recent years, more attention has been given to the role of ω-3 PUFAs in NAFLD by targeting the gut–liver axis. First, ω-3 PUFA direct exhibit an impact on the composition and diversity of gut microbiota [139]. For example, an endogenous generation of ω-3 PUFA by genetic approach resulted in lowered abundance of pro-inflammatory bacteria (e.g., *Proteobacteria*) and increased anti-inflammatory bacteria (e.g., *Bifidobacterium*, *Akkermansia muciniphila*) in the gut of mice, compared with wild-type littermates [140]. Secondly, dietary ω-3 PUFA supplementation elicits a decrease in plasma levels of endotoxin and TMA derived from bacteria. It has been reported that in contrast to the high concentration of serum endotoxin in response to saturated fat consumption, participants exposed to food with ω-3 PUFAs exhibited a reduction in endotoxin content [141]. Results from animal study have highlighted that DHA-enriched fish oil decreased the production of TMA and expedited TMAO metabolism through enhancing the activities of FMOs [142]. Thirdly, ω-3 PUFA contributes to the integrity of intestinal mucosa and to improvement of intestinal permeability as well as to the reduction of bacterial translocation, which has been reported in copious studies both in vivo and in vitro [143,144,145,146,147]. Lastly, ω-3 PUFAs produce a healthy bile acid reservoir and have been identified as FXR ligands, thereby conducing to the maintenance of liver health and the suppression of hepatic inflammation [50,148,149]. 

### 3.6. Carotenoids and Polyphenols

Food rich in carotenoids and polyphenols provides widespread benefits to humans and animals including gut and liver health. Carotenoids are a group of tetraterpenoids that mainly exist in plants, algae, fungi, and archaea [150]. One-month consumption of lycopene, the major carotenoid type in tomatoes, presented an increase in relative abundance of profitable strain *Bifidobacterium adolescentis* and *Bifidobacterium longum* in the gut of volunteers, along with an improvement of liver metabolism [151]. Serum level of carotenoids has been shown to be inversely correlated with the impaired intestinal barrier function [152]. Supplementation of carotenoids benefits to the gut barrier function, immune homeostasis, and dysbacteria through expediting immunoglobulin A (IgA) production in the gut [153,154]. Specific microbiota in the gut can be identified and enveloped by IgA, thereby making its translocation through the gut barrier hampered [155]. In addition to the protection against bacterial translocation, carotenoids have been known to provide anti-inflammatory and anti-oxidative effects. Results from animal models revealed the alleviative role of carotenoids on high-fat diet-induced NAFLD via attenuation of inflammatory response and oxidative stress [156,157,158]. The anti-inflammatory effect of carotenoids has also been ascertained in macrophages. For example, the inflammatory response in RAW264.7 macrophages or isolated primary macrophages from mice induced by LPS were shown to be mitigated by β-carotene, which was associated with the inactivation of nuclear factor-κB (NF-κB) and enhanced antioxidant capacity [159]. Although few reports support the regulation of carotenoids on bile acids, FXR, a palliative factor for hepatic steatosis, has been shown to be up-regulated in the liver of rats exposed to high-fat diet feeding with lycopene [160]. Polyphenols appertain to a highly diverse category of compounds that present naturally in plants [161]. Various polyphenols have been proved to display a protection effect on NAFLD by attenuation of insulin resistance, oxidative injury, and inflammation as well as by acceleration of fatty acid β-oxidation [162,163,164]. Clinical trial on volunteers presented ample evidence for reshaping gut microbiota by consumption of polyphenolic extract, leading to a reduction in the levels of serum TMAO [165]. Human study revealed that long-term strawberry polyphenols supplementation changed bile acid profiles and showed an impact on FXR/TGR5 signaling [166]. Polyphenols from berries and pomegranate fruit are metabolized by gut microbiota into urolithin A. This metabolite has been shown to possess anti-inflammatory and anti-oxidative properties and substantially boost intestinal barrier function [167], thus acting as a protector in chronic liver diseases including NAFLD. Additionally, polyphenols function to regulate intestinal barrier integrity. As detailed summarized by Yang et al. [168], a wide range of transcriptional factor, kinases, and enzymes correlate with the beneficial implications of polyphenols on intestinal barrier functions; therefore, these regulatory pathways may convey advantages to liver health.

## 4. Conclusions

**Figure 1:**
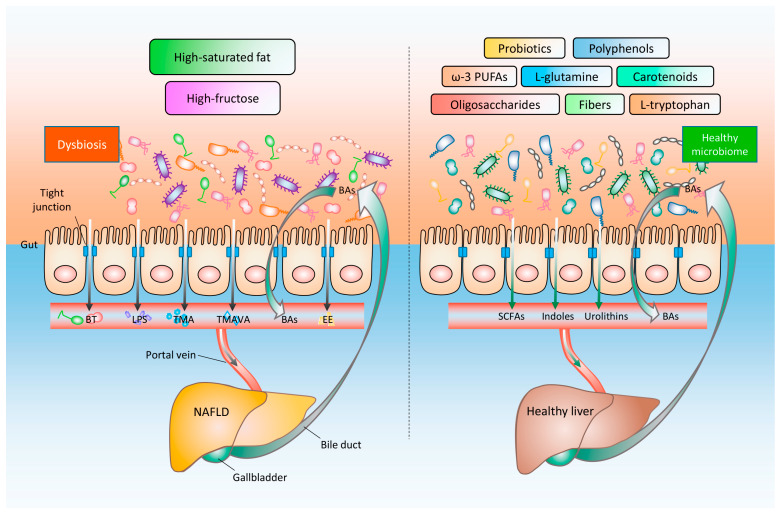
Non-alcoholic fatty liver disease (NAFLD) induced by unbalanced diet and its nutritional improvement strategies based on the gut–liver axis. Long-term high-saturated fat or high-fructose diet leads to an imbalanced intestinal flora, which in turn elicits an impaired gut barrier function and increased permeability, followed by bacterial translocation (BT), and additional bacterial components and metabolites (e.g., lipopolysaccharides (LPS), trimethylamine (TMA), N,N,N-trimethyl-5-aminovaleric acid (TMAVA), and endogenous ethanol (EE)) entering into the liver through the portal vein. NAFLD patients exhibit abnormal bile acids (BAs) metabolism and its related signaling pathways. These factors together accelerate the occurrence and progression of NAFLD. By contrast, an appropriate consumption of probiotics, functional oligosaccharides, dietary fibers, ω-3 polyunsaturated fatty acids (ω-3 PUFAs), functional amino acids (L-tryptophan and L-glutamine), carotenoids, and polyphenols, contributes (1) to the maintenance of the homeostasis of the intestinal flora and BAs, (2) to the enhancement of the intestinal barrier integrity, and (3) to the production of salutary metabolites (e.g., short chain fatty acids (SCFAs), indoles, and urolithins), thereby supporting a healthy liver.

In conclusion, the gut–liver axis has been a key component for the onset and progression of NAFLD. The exact mechanisms that connect gut microbiota with liver are complex and deserved additional thorough exploration. Intestinal flora perturbation and its concomitant pro-inflammatory initiators and disrupted bile acid homeostasis as well as gut barrier integrity have emerged as key regulators for metabolic dysfunction, leading to an acceleration of NAFLD progress. The targeting of gut–liver axis has been in the spotlight of metabolic diseases and may become imperative for the prevention and therapy of NAFLD in the future. The gut bacterial species serving to impact the gut–liver axis have been summarized in Table 1. Nutritional supplements are involved in the attenuation of NAFLD, since they facilitate the maintenance of homeostasis in gut microbiome, thereby improving the intestinal barrier function and bile acid profiles as well as reducing the migration of bacteria and harmful factors into liver (Figure 1). To provide explicit scientific evidence for the appropriate formulation of specific supplements for NAFLD patients, more attention needs to be paid to the comprehension of mechanisms whereby the beneficial effects of specific nutrients on the gut–liver axis. Following the specific characteristics of intestinal microbiome, the preparation of personalized dietary therapy offers a promising access to future NAFLD prevention and treatment.

## Figures and Tables

**Table 1:** The gut bacterial species that exhibit an impact on the gut–liver axis.

Gut Bacterial Species	Impact on the Gut–Liver Axis	References
*Enterobacter cloacae B29*, *Escherichia coli PY102*, and *Klebsiella pneumoniae A7*	Production of endotoxin	[42]
*Ruminococcus bromii*, *Ruminococcus gnavus*, and *Ruminococcus torques*	Breakdown of gut barrier function; Production of inflammatory polysaccharide; Increase of plasma TMAO	[169,170,171]
*Anaerococcus hydrogenalis*, *Clostridium asparagiforme*, *Clostridium hathewayi*, *Clostridium sporogenes*, *Escherichia fergusonii*, *Proteus penneri*, *Providencia rettgeri*, and *Edwardsiella tarda*	Generation of TMA Increase of plasma TMAO	[47]
*Enterococcus faecalis* and *Pseudomonas aeruginosa*	Production of TMAVA	[52]
*Escherichia* and *Klebsiella pneumoniae*	Production of endogenous ethanol	[41,64]
*Bifidobacterium* spp. and *Lactobacillus* spp.	Maintenance of intestinal barrier integrity	[169,172,173,174,175,176,177]
*Clostridium* and *Eubacterium*	Conversion of primary bile acids into secondary bile acids	[178]
*Clostridium* sp. *Strain S2*	Desulfation of bile acids; Promotion of bile acid reabsorption	[179]
*Eubacterium biforme*, *Prevotella copri*, *Ruminococcus torques*, *Fusobacterium*, and *Megashpaera*	Production of SCFAs	[180]
*Clostridium bartlettii*, *Clostridium sporogenes*, *Clostridium cadaveris*, *Clostridium botulinum*, *Bacteroides* spp., *Bifidobacterium* spp., *Lactobacillus* spp., and *Peptostreptococcus* spp.	Conversion of tryptophan into indoles	[115]
*Gordonibacter urolithinfaciens* and *Gordonibacter pamelaeae*	Generation of urolithins	[181]

## Abbreviations

AhR	aryl hydrocarbon receptor
DHA	Docosahexaenoic acid
EPA	eicosapentaenoic acid
FMO	flavin-containing monooxygenase
FXR	farnesoid X receptor
IBD	Inflammatory bowel disease
IAA	indole acetic acid
IAld	indole aldehyde
IA	indoleacrylic acid
ILA	indole lactic acid
IPA	indole propionic acid
LPS	lipopolysaccharides
NAFLD	non-alcoholic fatty liver disease
NASH	non-alcoholic steatohepatitis
PUFAs	polyunsaturated fatty acids
SCFAs	short chain fatty acids
TGR5	Takeda G-protein receptor 5
TLR4	toll-like receptor 4
TMA	trimethylamine
TMAO	trimethylamine-N-oxide
TMAVA	N,N,N-trimethyl-5-aminovaleric acid

## References

[1] Fabbrini E., Magkos F. (2015). Hepatic Steatosis as a Marker of Metabolic Dysfunction. Nutrients.

[2] Estes C., Razavi H., Loomba R., Younossi Z., Sanyal A.J. (2018). Modeling the epidemic of nonalcoholic fatty liver disease demonstrates an exponential increase in burden of disease. Hepatology.

[3] Buzzetti E., Pinzani M., Tsochatzis E.A. (2016). The multiple-hit pathogenesis of non-alcoholic fatty liver disease (NAFLD). Metabolism.

[4] Seko Y., Yamaguchi K., Itoh Y. (2018). The genetic backgrounds in nonalcoholic fatty liver disease. Clin. J. Gastroenterol..

[5] Eslam M., George J. (2020). Genetic contributions to NAFLD: Leveraging shared genetics to uncover systems biology. Nat. Rev. Gastroenterol. Hepatol..

[6] Del Campo J.A., Gallego-Duran R., Gallego P., Grande L. (2018). Genetic and Epigenetic Regulation in Nonalcoholic Fatty Liver Disease (NAFLD). Int. J. Mol. Sci..

[7] Guo X., Li J., Tang R., Zhang G., Zeng H., Wood R.J., Liu Z. (2017). High Fat Diet Alters Gut Microbiota and the Expression of Paneth Cell-Antimicrobial Peptides Preceding Changes of Circulating Inflammatory Cytokines. Mediat. Inflamm..

[8] Murphy E.A., Velazquez K.T., Herbert K.M. (2015). Influence of high-fat diet on gut microbiota: A driving force for chronic disease risk. Curr. Opin. Clin. Nutr. Metab. Care.

[9] Li J.M., Yu R., Zhang L.P., Wen S.Y., Wang S.J., Zhang X.Y., Xu Q., Kong L.D. (2019). Dietary fructose-induced gut dysbiosis promotes mouse hippocampal neuroinflammation: A benefit of short-chain fatty acids. Microbiome.

[10] Spruss A., Bergheim I. (2009). Dietary fructose and intestinal barrier: Potential risk factor in the pathogenesis of nonalcoholic fatty liver disease. J. Nutr. Biochem..

[11] Aron-Wisnewsky J., Vigliotti C., Witjes J., Le P., Holleboom A.G., Verheij J., Nieuwdorp M., Clement K. (2020). Gut microbiota and human NAFLD: Disentangling microbial signatures from metabolic disorders. Nat. Rev. Gastroenterol. Hepatol..

[12] Pitocco D., Di Leo M., Tartaglione L., De Leva F., Petruzziello C., Saviano A., Pontecorvi A., Ojetti V. (2020). The role of gut microbiota in mediating obesity and diabetes mellitus. Eur. Rev. Med. Pharm. Sci..

[13] Mokkala K., Houttu N., Cansev T., Laitinen K. (2019). Interactions of dietary fat with the gut microbiota: Evaluation of mechanisms and metabolic consequences. Clin. Nutr..

[14] Duranti S., Ferrario C., van Sinderen D., Ventura M., Turroni F. (2017). Obesity and microbiota: An example of an intricate relationship. Genes Nutr..

[15] Gerard P. (2016). Gut microbiota and obesity. Cell Mol. Life Sci..

[16] Sarwar R., Pierce N., Koppe S. (2018). Obesity and nonalcoholic fatty liver disease: Current perspectives. Diabetes Metab. Syndr. Obes..

[17] Polyzos S.A., Kountouras J., Mantzoros C.S. (2019). Obesity and nonalcoholic fatty liver disease: From pathophysiology to therapeutics. Metabolism.

[18] Boursier J., Rawls J.F., Diehl A.M. (2013). Obese humans with nonalcoholic fatty liver disease display alterations in fecal microbiota and volatile organic compounds. Clin. Gastroenterol. Hepatol..

[19] Wang B., Jiang X., Cao M., Ge J., Bao Q., Tang L., Chen Y., Li L. (2016). Altered Fecal Microbiota Correlates with Liver Biochemistry in Nonobese Patients with Non-alcoholic Fatty Liver Disease. Sci. Rep..

[20] Chiu C.C., Ching Y.H., Li Y.P., Liu J.Y., Huang Y.T., Huang Y.W., Yang S.S., Huang W.C., Chuang H.L. (2017). Nonalcoholic Fatty Liver Disease Is Exacerbated in High-Fat Diet-Fed Gnotobiotic Mice by Colonization with the Gut Microbiota from Patients with Nonalcoholic Steatohepatitis. Nutrients.

[21] Videhult F.K., West C.E. (2016). Nutrition, gut microbiota and child health outcomes. Curr. Opin. Clin. Nutr. Metab. Care.

[22] Singh R.K., Chang H.W., Yan D., Lee K.M., Ucmak D., Wong K., Abrouk M., Farahnik B., Nakamura M., Zhu T.H. (2017). Influence of diet on the gut microbiome and implications for human health. J. Transl. Med..

[23] De Santis S., Cavalcanti E., Mastronardi M., Jirillo E., Chieppa M. (2015). Nutritional Keys for Intestinal Barrier Modulation. Front. Immunol..

[24] Son G., Kremer M., Hines I.N. (2010). Contribution of gut bacteria to liver pathobiology. Gastroenterol. Res. Pr..

[25] Chiang J.Y. (2013). Bile acid metabolism and signaling. Compr. Physiol..

[26] Mendez-Salazar E.O., Ortiz-Lopez M.G., Granados-Silvestre M.L.A., Palacios-Gonzalez B., Menjivar M. (2018). Altered Gut Microbiota and Compositional Changes in Firmicutes and Proteobacteria in Mexican Undernourished and Obese Children. Front. Microbiol..

[27] Rajilic-Stojanovic M., de Vos W.M. (2014). The first 1000 cultured species of the human gastrointestinal microbiota. Fems Microbiol. Rev..

[28] Rinninella E., Raoul P., Cintoni M., Franceschi F., Miggiano G.A.D., Gasbarrini A., Mele M.C. (2019). What is the Healthy Gut Microbiota Composition? A Changing Ecosystem across Age, Environment, Diet, and Diseases. Microorganisms.

[29] Bastian W.P., Hasan I., Lesmana C.R.A., Rinaldi I., Gani R.A. (2019). Gut Microbiota Profiles in Nonalcoholic Fatty Liver Disease and Its Possible Impact on Disease Progression Evaluated with Transient Elastography: Lesson Learnt from 60 Cases. Case Rep. Gastroenterol..

[30] Mouzaki M., Comelli E.M., Arendt B.M., Bonengel J., Fung S.K., Fischer S.E., McGilvray I.D., Allard J.P. (2013). Intestinal microbiota in patients with nonalcoholic fatty liver disease. Hepatology.

[31] Wong V.W., Tse C.H., Lam T.T., Wong G.L., Chim A.M., Chu W.C., Yeung D.K., Law P.T., Kwan H.S., Yu J. (2013). Molecular characterization of the fecal microbiota in patients with nonalcoholic steatohepatitis—A longitudinal study. PLoS ONE.

[32] Sobhonslidsuk A., Chanprasertyothin S., Pongrujikorn T., Kaewduang P., Promson K., Petraksa S., Ongphiphadhanakul B. (2018). The Association of Gut Microbiota with Nonalcoholic Steatohepatitis in Thais. Biomed. Res. Int..

[33] Boursier J., Mueller O., Barret M., Machado M., Fizanne L., Araujo-Perez F., Guy C.D., Seed P.C., Rawls J.F., David L.A. (2016). The severity of nonalcoholic fatty liver disease is associated with gut dysbiosis and shift in the metabolic function of the gut microbiota. Hepatology.

[34] Loomba R., Seguritan V., Li W., Long T., Klitgord N., Bhatt A., Dulai P.S., Caussy C., Bettencourt R., Highlander S.K. (2017). Gut Microbiome-Based Metagenomic Signature for Non-invasive Detection of Advanced Fibrosis in Human Nonalcoholic Fatty Liver Disease. Cell Metab..

[35] Frazier T.H., DiBaise J.K., McClain C.J. (2011). Gut microbiota, intestinal permeability, obesity-induced inflammation, and liver injury. Jpen J. Parenter. Enter. Nutr..

[36] Haukeland J.W., Damas J.K., Konopski Z., Loberg E.M., Haaland T., Goverud I., Torjesen P.A., Birkeland K., Bjoro K., Aukrust P. (2006). Systemic inflammation in nonalcoholic fatty liver disease is characterized by elevated levels of CCL2. J. Hepatol..

[37] Tyrovolas S., Panagiotakos D.B., Georgousopoulou E.N., Chrysohoou C., Skoumas J., Pan W., Tousoulis D., Pitsavos C. (2019). The anti-inflammatory potential of diet and nonalcoholic fatty liver disease: The ATTICA study. Ther. Adv. Gastroenterol..

[38] Wong V.W., Wong G.L., Chan H.Y., Yeung D.K., Chan R.S., Chim A.M., Chan C.K., Tse Y.K., Woo J., Chu W.C. (2015). Bacterial endotoxin and non-alcoholic fatty liver disease in the general population: A prospective cohort study. Aliment. Pharm..

[39] Harte A.L., da Silva N.F., Creely S.J., McGee K.C., Billyard T., Youssef-Elabd E.M., Tripathi G., Ashour E., Abdalla M.S., Sharada H.M. (2010). Elevated endotoxin levels in non-alcoholic fatty liver disease. J. Inflamm. (Lond.).

[40] Mannisto V., Farkkila M., Pussinen P., Jula A., Mannisto S., Lundqvist A., Valsta L., Salomaa V., Perola M., Aberg F. (2019). Serum lipopolysaccharides predict advanced liver disease in the general population. Jhep Rep..

[41] Zhu L., Baker S.S., Gill C., Liu W., Alkhouri R., Baker R.D., Gill S.R. (2013). Characterization of gut microbiomes in nonalcoholic steatohepatitis (NASH) patients: A connection between endogenous alcohol and NASH. Hepatology.

[42] Fei N., Bruneau A., Zhang X., Wang R., Wang J., Rabot S., Gerard P., Zhao L. (2020). Endotoxin Producers Overgrowing in Human Gut Microbiota as the Causative Agents for Nonalcoholic Fatty Liver Disease. mBio.

[43] Bibbo S., Ianiro G., Dore M.P., Simonelli C., Newton E.E., Cammarota G. (2018). Gut Microbiota as a Driver of Inflammation in Nonalcoholic Fatty Liver Disease. Mediat. Inflamm..

[44] Lu Y.C., Yeh W.C., Ohashi P.S. (2008). LPS/TLR4 signal transduction pathway. Cytokine.

[45] Ji Y., Yin Y., Li Z., Zhang W. (2019). Gut Microbiota-Derived Components and Metabolites in the Progression of Non-Alcoholic Fatty Liver Disease (NAFLD). Nutrients.

[46] Barrea L., Annunziata G., Muscogiuri G., Di Somma C., Laudisio D., Maisto M., de Alteriis G., Tenore G.C., Colao A., Savastano S. (2018). Trimethylamine-N-oxide (TMAO) as Novel Potential Biomarker of Early Predictors of Metabolic Syndrome. Nutrients.

[47] Romano K.A., Vivas E.I., Amador-Noguez D., Rey F.E. (2015). Intestinal microbiota composition modulates choline bioavailability from diet and accumulation of the proatherogenic metabolite trimethylamine-N-oxide. mBio.

[48] Canyelles M., Tondo M., Cedo L., Farras M., Escola-Gil J.C., Blanco-Vaca F. (2018). Trimethylamine N-Oxide: A Link among Diet, Gut Microbiota, Gene Regulation of Liver and Intestine Cholesterol Homeostasis and HDL Function. Int. J. Mol. Sci..

[49] Tan X., Liu Y., Long J., Chen S., Liao G., Wu S., Li C., Wang L., Ling W., Zhu H. (2019). Trimethylamine N-Oxide Aggravates Liver Steatosis through Modulation of Bile Acid Metabolism and Inhibition of Farnesoid X Receptor Signaling in Nonalcoholic Fatty Liver Disease. Mol. Nutr. Food Res..

[50] Armstrong L.E., Guo G.L. (2017). Role of FXR in Liver Inflammation during Nonalcoholic Steatohepatitis. Curr. Pharm. Rep..

[51] Zheng Z., Zhao Z., Li S., Lu X., Jiang M., Lin J., An Y., Xie Y., Xu M., Shen W. (2017). Altenusin, a Nonsteroidal Microbial Metabolite, Attenuates Nonalcoholic Fatty Liver Disease by Activating the Farnesoid X Receptor. Mol. Pharm..

[52] Zhao M., Zhao L., Xiong X., He Y., Huang W., Liu Z., Ji L., Pan B., Guo X., Wang L. (2020). TMAVA, a Metabolite of Intestinal Microbes, Is Increased in Plasma From Patients With Liver Steatosis, Inhibits gamma-butyrobetaine Hydroxylase, and Exacerbates Fatty Liver in Mice. Gastroenterology.

[53] Vancamelbeke M., Vermeire S. (2017). The intestinal barrier: A fundamental role in health and disease. Expert Rev. Gastroenterol. Hepatol..

[54] Zeng T., Zhang C.L., Xiao M., Yang R., Xie K.Q. (2016). Critical Roles of Kupffer Cells in the Pathogenesis of Alcoholic Liver Disease: From Basic Science to Clinical Trials. Front. Immunol..

[55] Miele L., Valenza V., La Torre G., Montalto M., Cammarota G., Ricci R., Masciana R., Forgione A., Gabrieli M.L., Perotti G. (2009). Increased intestinal permeability and tight junction alterations in nonalcoholic fatty liver disease. Hepatology.

[56] Belei O., Olariu L., Dobrescu A., Marcovici T., Marginean O. (2017). The relationship between non-alcoholic fatty liver disease and small intestinal bacterial overgrowth among overweight and obese children and adolescents. J. Pediatr. Endocrinol. Metab..

[57] Ilan Y. (2012). Leaky gut and the liver: A role for bacterial translocation in nonalcoholic steatohepatitis. World J. Gastroenterol..

[58] Sartini A., Gitto S., Bianchini M., Verga M.C., Di Girolamo M., Bertani A., Del Buono M., Schepis F., Lei B., De Maria N. (2018). Non-alcoholic fatty liver disease phenotypes in patients with inflammatory bowel disease. Cell Death Dis..

[59] Mouries J., Brescia P., Silvestri A., Spadoni I., Sorribas M., Wiest R., Mileti E., Galbiati M., Invernizzi P., Adorini L. (2019). Microbiota-driven gut vascular barrier disruption is a prerequisite for non-alcoholic steatohepatitis development. J. Hepatol..

[60] Fasano A. (2012). Intestinal permeability and its regulation by zonulin: Diagnostic and therapeutic implications. Clin. Gastroenterol. Hepatol..

[61] Li C., Gao M., Zhang W., Chen C., Zhou F., Hu Z., Zeng C. (2016). Zonulin Regulates Intestinal Permeability and Facilitates Enteric Bacteria Permeation in Coronary Artery Disease. Sci. Rep..

[62] Hendy O.M., Elsabaawy M.M., Aref M.M., Khalaf F.M., Oda A.M.A., El Shazly H.M. (2017). Evaluation of circulating zonulin as a potential marker in the pathogenesis of nonalcoholic fatty liver disease. APMIS.

[63] de Medeiros I.C., de Lima J.G. (2015). Is nonalcoholic fatty liver disease an endogenous alcoholic fatty liver disease? A mechanistic hypothesis. Med. Hypotheses.

[64] Yuan J., Chen C., Cui J., Lu J., Yan C., Wei X., Zhao X., Li N., Li S., Xue G. (2019). Fatty Liver Disease Caused by High-Alcohol-Producing Klebsiella pneumoniae. Cell Metab..

[65] Sarenac T.M., Mikov M. (2018). Bile Acid Synthesis: From Nature to the Chemical Modification and Synthesis and Their Applications as Drugs and Nutrients. Front. Pharm..

[66] de Aguiar Vallim T.Q., Tarling E.J., Edwards P.A. (2013). Pleiotropic roles of bile acids in metabolism. Cell Metab..

[67] Staley C., Weingarden A.R., Khoruts A., Sadowsky M.J. (2017). Interaction of gut microbiota with bile acid metabolism and its influence on disease states. Appl. Microbiol. Biotechnol..

[68] Wahlstrom A., Sayin S.I., Marschall H.U., Backhed F. (2016). Intestinal Crosstalk between Bile Acids and Microbiota and Its Impact on Host Metabolism. Cell Metab..

[69] Ridlon J.M., Harris S.C., Bhowmik S., Kang D.J., Hylemon P.B. (2016). Consequences of bile salt biotransformations by intestinal bacteria. Gut Microbes.

[70] Cai J.S., Chen J.H. (2014). The mechanism of enterohepatic circulation in the formation of gallstone disease. J. Membr. Biol..

[71] Kim H., Fang S. (2018). Crosstalk between FXR and TGR5 controls glucagon-like peptide 1 secretion to maintain glycemic homeostasis. Lab. Anim. Res..

[72] Chai J., Zou L., Li X., Han D., Wang S., Hu S., Guan J. (2015). Mechanism of bile acid-regulated glucose and lipid metabolism in duodenal-jejunal bypass. Int. J. Clin. Exp. Pathol..

[73] Iracheta-Vellve A., Calenda C.D., Petrasek J., Ambade A., Kodys K., Adorini L., Szabo G. (2018). FXR and TGR5 Agonists Ameliorate Liver Injury, Steatosis, and Inflammation After Binge or Prolonged Alcohol Feeding in Mice. Hepatol. Commun..

[74] Chiang J.Y., Pathak P., Liu H., Donepudi A., Ferrell J., Boehme S. (2017). Intestinal Farnesoid X Receptor and Takeda G Protein Couple Receptor 5 Signaling in Metabolic Regulation. Dig. Dis..

[75] Chiang J.Y. (2015). Negative feedback regulation of bile acid metabolism: Impact on liver metabolism and diseases. Hepatology.

[76] Wang C., Zhu C., Shao L., Ye J., Shen Y., Ren Y. (2019). Role of Bile Acids in Dysbiosis and Treatment of Nonalcoholic Fatty Liver Disease. Mediat. Inflamm..

[77] Kalhan S.C., Guo L., Edmison J., Dasarathy S., McCullough A.J., Hanson R.W., Milburn M. (2011). Plasma metabolomic profile in nonalcoholic fatty liver disease. Metabolism.

[78] Chiang J.Y.L., Ferrell J.M. (2020). Bile acid receptors FXR and TGR5 signaling in fatty liver diseases and therapy. Am. J. Physiol. Gastrointest. Liver Physiol..

[79] Finn P.D., Rodriguez D., Kohler J., Jiang Z., Wan S., Blanco E., King A.J., Chen T., Bell N., Dragoli D. (2019). Intestinal TGR5 agonism improves hepatic steatosis and insulin sensitivity in Western diet-fed mice. Am. J. Physiol. Gastrointest. Liver Physiol..

[80] Hill C., Guarner F., Reid G., Gibson G.R., Merenstein D.J., Pot B., Morelli L., Canani R.B., Flint H.J., Salminen S. (2014). Expert consensus document. The International Scientific Association for Probiotics and Prebiotics consensus statement on the scope and appropriate use of the term probiotic. Nat. Rev. Gastroenterol. Hepatol..

[81] Woo J., Ahn J. (2013). Probiotic-mediated competition, exclusion and displacement in biofilm formation by food-borne pathogens. Lett. Appl. Microbiol..

[82] Corthesy B., Gaskins H.R., Mercenier A. (2007). Cross-talk between probiotic bacteria and the host immune system. J. Nutr..

[83] Ritze Y., Bardos G., Claus A., Ehrmann V., Bergheim I., Schwiertz A., Bischoff S.C. (2014). Lactobacillus rhamnosus GG protects against non-alcoholic fatty liver disease in mice. PLoS ONE.

[84] Jang H.R., Park H.J., Kang D., Chung H., Nam M.H., Lee Y., Park J.H., Lee H.Y. (2019). A protective mechanism of probiotic Lactobacillus against hepatic steatosis via reducing host intestinal fatty acid absorption. Exp. Mol. Med..

[85] Ling X., Linglong P., Weixia D., Hong W. (2016). Protective Effects of Bifidobacterium on Intestinal Barrier Function in LPS-Induced Enterocyte Barrier Injury of Caco-2 Monolayers and in a Rat NEC Model. PLoS ONE.

[86] Kobyliak N., Abenavoli L., Mykhalchyshyn G., Kononenko L., Boccuto L., Kyriienko D., Dynnyk O. (2018). A Multi-strain Probiotic Reduces the Fatty Liver Index, Cytokines and Aminotransferase levels in NAFLD Patients: Evidence from a Randomized Clinical Trial. J. Gastrointestin. Liver Dis..

[87] Boutagy N.E., Neilson A.P., Osterberg K.L., Smithson A.T., Englund T.R., Davy B.M., Hulver M.W., Davy K.P. (2015). Probiotic supplementation and trimethylamine-N-oxide production following a high-fat diet. Obes. (Silver Spring).

[88] Tripolt N.J., Leber B., Triebl A., Kofeler H., Stadlbauer V., Sourij H. (2015). Effect of Lactobacillus casei Shirota supplementation on trimethylamine-N-oxide levels in patients with metabolic syndrome: An open-label, randomized study. Atherosclerosis.

[89] Culpepper T., Rowe C.C., Rusch C.T., Burns A.M., Federico A.P., Girard S.A., Tompkins T.A., Nieves C., Dennis-Wall J.C., Christman M.C. (2019). Three probiotic strains exert different effects on plasma bile acid profiles in healthy obese adults: Randomised, double-blind placebo-controlled crossover study. Benef. Microbes.

[90] Degirolamo C., Rainaldi S., Bovenga F., Murzilli S., Moschetta A. (2014). Microbiota modification with probiotics induces hepatic bile acid synthesis via downregulation of the Fxr-Fgf15 axis in mice. Cell Rep..

[91] Cui G., Martin R.C., Jin H., Liu X., Pandit H., Zhao H., Cai L., Zhang P., Li W., Li Y. (2018). Up-regulation of FGF15/19 signaling promotes hepatocellular carcinoma in the background of fatty liver. J. Exp. Clin. Cancer Res..

[92] Schumacher J.D., Kong B., Pan Y., Zhan L., Sun R., Aa J., Rizzolo D., Richardson J.R., Chen A., Goedken M. (2017). The effect of fibroblast growth factor 15 deficiency on the development of high fat diet induced non-alcoholic steatohepatitis. Toxicol. Appl. Pharm..

[93] Stephen A.M., Champ M.M., Cloran S.J., Fleith M., van Lieshout L., Mejborn H., Burley V.J. (2017). Dietary fibre in Europe: Current state of knowledge on definitions, sources, recommendations, intakes and relationships to health. Nutr. Res. Rev..

[94] Jones J.M. (2013). Dietary fiber future directions: Integrating new definitions and findings to inform nutrition research and communication. Adv. Nutr..

[95] Zolfaghari H., Askari G., Siassi F., Feizi A., Sotoudeh G. (2016). Intake of Nutrients, Fiber, and Sugar in Patients with Nonalcoholic Fatty Liver Disease in Comparison to Healthy Individuals. Int. J. Prev. Med..

[96] Kim C.H., Kallman J.B., Bai C., Pawloski L., Gewa C., Arsalla A., Sabatella M.E., Younossi Z.M. (2010). Nutritional assessments of patients with non-alcoholic fatty liver disease. Obes. Surg..

[97] Xia Y., Zhang S., Zhang Q., Liu L., Meng G., Wu H., Bao X., Gu Y., Sun S., Wang X. (2020). Insoluble dietary fibre intake is associated with lower prevalence of newly-diagnosed non-alcoholic fatty liver disease in Chinese men: A large population-based cross-sectional study. Nutr. Metab. (Lond.).

[98] Holscher H.D. (2017). Dietary fiber and prebiotics and the gastrointestinal microbiota. Gut Microbes.

[99] Tan J., McKenzie C., Potamitis M., Thorburn A.N., Mackay C.R., Macia L. (2014). The role of short-chain fatty acids in health and disease. Adv. Immunol..

[100] Singh J., Metrani R., Shivanagoudra S.R., Jayaprakasha G.K., Patil B.S. (2019). Review on Bile Acids: Effects of the Gut Microbiome, Interactions with Dietary Fiber, and Alterations in the Bioaccessibility of Bioactive Compounds. J. Agric. Food Chem..

[101] Naumann S., Schweiggert-Weisz U., Eglmeier J., Haller D., Eisner P. (2019). In Vitro Interactions of Dietary Fibre Enriched Food Ingredients with Primary and Secondary Bile Acids. Nutrients.

[102] Cantero I., Abete I., Monreal J.I., Martinez J.A., Zulet M.A. (2017). Fruit Fiber Consumption Specifically Improves Liver Health Status in Obese Subjects under Energy Restriction. Nutrients.

[103] Yang L., Zhao Y., Huang J., Zhang H., Lin Q., Han L., Liu J., Wang J., Liu H. (2020). Insoluble dietary fiber from soy hulls regulates the gut microbiota in vitro and increases the abundance of bifidobacteriales and lactobacillales. J. Food Sci. Technol..

[104] Krawczyk M., Maciejewska D., Ryterska K., Czerwinka-Rogowska M., Jamiol-Milc D., Skonieczna-Zydecka K., Milkiewicz P., Raszeja-Wyszomirska J., Stachowska E. (2018). Gut Permeability Might be Improved by Dietary Fiber in Individuals with Nonalcoholic Fatty Liver Disease (NAFLD) Undergoing Weight Reduction. Nutrients.

[105] Pan L., Farouk M.H., Qin G., Zhao Y., Bao N. (2018). The Influences of Soybean Agglutinin and Functional Oligosaccharides on the Intestinal Tract of Monogastric Animals. Int. J. Mol. Sci..

[106] Bai Y., Zheng J., Yuan X., Jiao S., Feng C., Du Y., Liu H., Zheng L. (2018). Chitosan Oligosaccharides Improve Glucolipid Metabolism Disorder in Liver by Suppression of Obesity-Related Inflammation and Restoration of Peroxisome Proliferator-Activated Receptor Gamma (PPARgamma). Mar. Drugs.

[107] Matsumoto K., Ichimura M., Tsuneyama K., Moritoki Y., Tsunashima H., Omagari K., Hara M., Yasuda I., Miyakawa H., Kikuchi K. (2017). Fructo-oligosaccharides and intestinal barrier function in a methionine-choline-deficient mouse model of nonalcoholic steatohepatitis. PLoS ONE.

[108] Cani P.D., Neyrinck A.M., Fava F., Knauf C., Burcelin R.G., Tuohy K.M., Gibson G.R., Delzenne N.M. (2007). Selective increases of bifidobacteria in gut microflora improve high-fat-diet-induced diabetes in mice through a mechanism associated with endotoxaemia. Diabetologia.

[109] Qian M., Lyu Q., Liu Y., Hu H., Wang S., Pan C., Duan X., Gao Y., Qi L.W., Liu W. (2019). Chitosan Oligosaccharide Ameliorates Nonalcoholic Fatty Liver Disease (NAFLD) in Diet-Induced Obese Mice. Mar. Drugs.

[110] Hoving L.R., Katiraei S., Heijink M., Pronk A., van der Wee-Pals L., Streefland T., Giera M., Willems van Dijk K., van Harmelen V. (2018). Dietary Mannan Oligosaccharides Modulate Gut Microbiota, Increase Fecal Bile Acid Excretion, and Decrease Plasma Cholesterol and Atherosclerosis Development. Mol. Nutr. Food Res..

[111] van Meer H., Boehm G., Stellaard F., Vriesema A., Knol J., Havinga R., Sauer P.J., Verkade H.J. (2008). Prebiotic oligosaccharides and the enterohepatic circulation of bile salts in rats. Am. J. Physiol. Gastrointest. Liver Physiol..

[112] Le Bourgot C., Apper E., Blat S., Respondek F. (2018). Fructo-oligosaccharides and glucose homeostasis: A systematic review and meta-analysis in animal models. Nutr. Metab. (Lond.).

[113] Jenkins T.A., Nguyen J.C., Polglaze K.E., Bertrand P.P. (2016). Influence of Tryptophan and Serotonin on Mood and Cognition with a Possible Role of the Gut-Brain Axis. Nutrients.

[114] Gostner J.M., Geisler S., Stonig M., Mair L., Sperner-Unterweger B., Fuchs D. (2020). Tryptophan Metabolism and Related Pathways in Psychoneuroimmunology: The Impact of Nutrition and Lifestyle. Neuropsychobiology.

[115] Roager H.M., Licht T.R. (2018). Microbial tryptophan catabolites in health and disease. Nat. Commun..

[116] Hou Q., Ye L., Liu H., Huang L., Yang Q., Turner J.R., Yu Q. (2018). Lactobacillus accelerates ISCs regeneration to protect the integrity of intestinal mucosa through activation of STAT3 signaling pathway induced by LPLs secretion of IL-22. Cell Death Differ..

[117] Ma L., Li H., Hu J., Zheng J., Zhou J., Botchlett R., Matthews D., Zeng T., Chen L., Xiao X. (2020). Indole Alleviates Diet-induced Hepatic Steatosis and Inflammation in a Manner Involving Myeloid Cell PFKFB3. Hepatology.

[118] Zhao Z.H., Xin F.Z., Xue Y., Hu Z., Han Y., Ma F., Zhou D., Liu X.L., Cui A., Liu Z. (2019). Indole-3-propionic acid inhibits gut dysbiosis and endotoxin leakage to attenuate steatohepatitis in rats. Exp. Mol. Med..

[119] Ji Y., Gao Y., Chen H., Yin Y., Zhang W. (2019). Indole-3-Acetic Acid Alleviates Nonalcoholic Fatty Liver Disease in Mice via Attenuation of Hepatic Lipogenesis, and Oxidative and Inflammatory Stress. Nutrients.

[120] Ji Y., Yin W., Liang Y., Sun L., Yin Y., Zhang W. (2020). Anti-Inflammatory and Anti-Oxidative Activity of Indole-3-Acetic Acid Involves Induction of HO-1 and Neutralization of Free Radicals in RAW264.7 Cells. Int. J. Mol. Sci..

[121] Sakurai T., Odamaki T., Xiao J.Z. (2019). Production of Indole-3-Lactic Acid by Bifidobacterium Strains Isolated fromHuman Infants. Microorganisms.

[122] Cruzat V., Macedo Rogero M., Noel Keane K., Curi R., Newsholme P. (2018). Glutamine: Metabolism and Immune Function, Supplementation and Clinical Translation. Nutrients.

[123] Wu G., Fang Y.Z., Yang S., Lupton J.R., Turner N.D. (2004). Glutathione metabolism and its implications for health. J. Nutr..

[124] Rao R., Samak G. (2012). Role of Glutamine in Protection of Intestinal Epithelial Tight Junctions. J. Epithel. Biol. Pharm..

[125] Wang B., Wu G., Zhou Z., Dai Z., Sun Y., Ji Y., Li W., Wang W., Liu C., Han F. (2015). Glutamine and intestinal barrier function. Amino Acids.

[126] de Souza A.Z., Zambom A.Z., Abboud K.Y., Reis S.K., Tannihao F., Guadagnini D., Saad M.J., Prada P.O. (2015). Oral supplementation with L-glutamine alters gut microbiota of obese and overweight adults: A pilot study. Nutrition.

[127] Sellmann C., Baumann A., Brandt A., Jin C.J., Nier A., Bergheim I. (2017). Oral Supplementation of Glutamine Attenuates the Progression of Nonalcoholic Steatohepatitis in C57BL/6J Mice. J. Nutr..

[128] Calder P.C. (2013). Omega-3 polyunsaturated fatty acids and inflammatory processes: Nutrition or pharmacology?. Br. J. Clin. Pharm..

[129] Brown T.J., Brainard J., Song F., Wang X., Abdelhamid A., Hooper L., Group P. (2019). Omega-3, omega-6, and total dietary polyunsaturated fat for prevention and treatment of type 2 diabetes mellitus: Systematic review and meta-analysis of randomised controlled trials. BMJ.

[130] Cleland L.G., James M.J., Proudman S.M. (2003). Omega-6/omega-3 fatty acids and arthritis. World Rev. Nutr. Diet..

[131] Simopoulos A.P. (2008). The importance of the omega-6/omega-3 fatty acid ratio in cardiovascular disease and other chronic diseases. Exp. Biol. Med. (Maywood).

[132] Simopoulos A.P. (2016). An Increase in the Omega-6/Omega-3 Fatty Acid Ratio Increases the Risk for Obesity. Nutrients.

[133] Simopoulos A.P., DiNicolantonio J.J. (2017). Mediterranean diet: Omega-6 and omega-3 fatty acids and diabetes. Am. J. Clin. Nutr..

[134] He X.X., Wu X.L., Chen R.P., Chen C., Liu X.G., Wu B.J., Huang Z.M. (2016). Effectiveness of Omega-3 Polyunsaturated Fatty Acids in Non-Alcoholic Fatty Liver Disease: A Meta-Analysis of Randomized Controlled Trials. PLoS ONE.

[135] Yan J.H., Guan B.J., Gao H.Y., Peng X.E. (2018). Omega-3 polyunsaturated fatty acid supplementation and non-alcoholic fatty liver disease: A meta-analysis of randomized controlled trials. Medicine.

[136] Lu W., Li S., Li J., Wang J., Zhang R., Zhou Y., Yin Q., Zheng Y., Wang F., Xia Y. (2016). Effects of Omega-3 Fatty Acid in Nonalcoholic Fatty Liver Disease: A Meta-Analysis. Gastroenterol. Res. Pr..

[137] Yuan F., Wang H., Tian Y., Li Q., He L., Li N., Liu Z. (2016). Fish oil alleviated high-fat diet-induced non-alcoholic fatty liver disease via regulating hepatic lipids metabolism and metaflammation: A transcriptomic study. Lipids Health Dis..

[138] de Castro G.S., Calder P.C. (2018). Non-alcoholic fatty liver disease and its treatment with n-3 polyunsaturated fatty acids. Clin. Nutr..

[139] Costantini L., Molinari R., Farinon B., Merendino N. (2017). Impact of Omega-3 Fatty Acids on the Gut Microbiota. Int. J. Mol. Sci..

[140] Kaliannan K., Wang B., Li X.Y., Kim K.J., Kang J.X. (2015). A host-microbiome interaction mediates the opposing effects of omega-6 and omega-3 fatty acids on metabolic endotoxemia. Sci. Rep..

[141] Lyte J.M., Gabler N.K., Hollis J.H. (2016). Postprandial serum endotoxin in healthy humans is modulated by dietary fat in a randomized, controlled, cross-over study. Lipids Health Dis..

[142] Yu J., Zhang T., Gao X., Xue C., Xu J., Wang Y. (2017). Fish oil affects the metabolic process of trimethylamine N-oxide precursor through trimethylamine production and flavin-containing monooxygenase activity in male C57BL/6 mice. Rsc Adv..

[143] Li Q., Zhang Q., Wang M., Zhao S., Xu G., Li J. (2008). n-3 polyunsaturated fatty acids prevent disruption of epithelial barrier function induced by proinflammatory cytokines. Mol. Immunol..

[144] Liu Y., Chen F., Odle J., Lin X., Jacobi S.K., Zhu H., Wu Z., Hou Y. (2012). Fish oil enhances intestinal integrity and inhibits TLR4 and NOD2 signaling pathways in weaned pigs after LPS challenge. J. Nutr..

[145] Li Q., Zhang Q., Wang C., Tang C., Zhang Y., Li N., Li J. (2011). Fish oil enhances recovery of intestinal microbiota and epithelial integrity in chronic rejection of intestinal transplant. PLoS ONE.

[146] Xiao G., Tang L., Yuan F., Zhu W., Zhang S., Liu Z., Geng Y., Qiu X., Zhang Y., Su L. (2013). Eicosapentaenoic acid enhances heat stress-impaired intestinal epithelial barrier function in Caco-2 cells. PLoS ONE.

[147] Generoso Sde V., Rodrigues N.M., Trindade L.M., Paiva N.C., Cardoso V.N., Carneiro C.M., Ferreira A.V., Faria A.M., Maioli T.U. (2015). Dietary supplementation with omega-3 fatty acid attenuates 5-fluorouracil induced mucositis in mice. Lipids Health Dis..

[148] Cieslak A., Trottier J., Verreault M., Milkiewicz P., Vohl M.C., Barbier O. (2018). N-3 Polyunsaturated Fatty Acids Stimulate Bile Acid Detoxification in Human Cell Models. Can. J. Gastroenterol. Hepatol..

[149] Zhao A., Yu J., Lew J.L., Huang L., Wright S.D., Cui J. (2004). Polyunsaturated fatty acids are FXR ligands and differentially regulate expression of FXR targets. DNA Cell Biol..

[150] Maoka T. (2020). Carotenoids as natural functional pigments. J. Nat. Med..

[151] Wiese M., Bashmakov Y., Chalyk N., Nielsen D.S., Krych L., Kot W., Klochkov V., Pristensky D., Bandaletova T., Chernyshova M. (2019). Prebiotic Effect of Lycopene and Dark Chocolate on Gut Microbiome with Systemic Changes in Liver Metabolism, Skeletal Muscles and Skin in Moderately Obese Persons. Biomed. Res. Int..

[152] Vieira M.M., Paik J., Blaner W.S., Soares A.M., Mota R.M., Guerrant R.L., Lima A.A. (2008). Carotenoids, retinol, and intestinal barrier function in children from northeastern Brazil. J. Pediatr. Gastroenterol. Nutr..

[153] Lyu Y., Wu L., Wang F., Shen X., Lin D. (2018). Carotenoid supplementation and retinoic acid in immunoglobulin A regulation of the gut microbiota dysbiosis. Exp. Biol. Med..

[154] Wells J.M., Brummer R.J., Derrien M., MacDonald T.T., Troost F., Cani P.D., Theodorou V., Dekker J., Meheust A., de Vos W.M. (2017). Homeostasis of the gut barrier and potential biomarkers. Am. J. Physiol. Gastrointest. Liver Physiol..

[155] Maes M., Kubera M., Leunis J.C., Berk M. (2012). Increased IgA and IgM responses against gut commensals in chronic depression: Further evidence for increased bacterial translocation or leaky gut. J. Affect. Disord..

[156] Bahcecioglu I.H., Kuzu N., Metin K., Ozercan I.H., Ustundag B., Sahin K., Kucuk O. (2010). Lycopene prevents development of steatohepatitis in experimental nonalcoholic steatohepatitis model induced by high-fat diet. Vet. Med. Int..

[157] Kobori M., Ni Y., Takahashi Y., Watanabe N., Sugiura M., Ogawa K., Nagashimada M., Kaneko S., Naito S., Ota T. (2014). beta-Cryptoxanthin alleviates diet-induced nonalcoholic steatohepatitis by suppressing inflammatory gene expression in mice. PLoS ONE.

[158] Seif El-Din S.H., El-Lakkany N.M., El-Naggar A.A., Hammam O.A., Abd El-Latif H.A., Ain-Shoka A.A., Ebeid F.A. (2015). Effects of rosuvastatin and/or beta-carotene on non-alcoholic fatty liver in rats. Res. Pharm. Sci..

[159] Bai S.K., Lee S.J., Na H.J., Ha K.S., Han J.A., Lee H., Kwon Y.G., Chung C.K., Kim Y.M. (2005). beta-Carotene inhibits inflammatory gene expression in lipopolysaccharide-stimulated macrophages by suppressing redox-based NF-kappaB activation. Exp. Mol. Med..

[160] Elvira-Torales L.I., Navarro-Gonzalez I., Gonzalez-Barrio R., Martin-Pozuelo G., Domenech G., Seva J., Garcia-Alonso J., Periago-Caston M.J. (2018). Tomato Juice Supplementation Influences the Gene Expression Related to Steatosis in Rats. Nutrients.

[161] Tsao R. (2010). Chemistry and biochemistry of dietary polyphenols. Nutrients.

[162] Musolino V., Gliozzi M., Scarano F., Bosco F., Scicchitano M., Nucera S., Carresi C., Ruga S., Zito M.C., Maiuolo J. (2020). Bergamot Polyphenols Improve Dyslipidemia and Pathophysiological Features in a Mouse Model of Non-Alcoholic Fatty Liver Disease. Sci. Rep..

[163] Van De Wier B., Koek G.H., Bast A., Haenen G.R. (2017). The potential of flavonoids in the treatment of non-alcoholic fatty liver disease. Crit. Rev. Food Sci. Nutr..

[164] Rafiei H., Omidian K., Bandy B. (2019). Dietary Polyphenols Protect Against Oleic Acid-Induced Steatosis in an in Vitro Model of NAFLD by Modulating Lipid Metabolism and Improving Mitochondrial Function. Nutrients.

[165] Annunziata G., Maisto M., Schisano C., Ciampaglia R., Narciso V., Tenore G.C., Novellino E. (2019). Effects of Grape Pomace Polyphenolic Extract (Taurisolo((R))) in Reducing TMAO Serum Levels in Humans: Preliminary Results from a Randomized, Placebo-Controlled, Cross-Over Study. Nutrients.

[166] Zhao A., Zhang X., Sandhu A., Edirisinghe I., Shukitt-Hale B., Burton-Freeman B. (2019). Polyphenol Consumption on Human Bile Acids Metabolism: Preliminary Data of Bile Acid Profiles in Human Biological Samples. Curr. Dev. Nutr..

[167] Singh R., Chandrashekharappa S., Bodduluri S.R., Baby B.V., Hegde B., Kotla N.G., Hiwale A.A., Saiyed T., Patel P., Vijay-Kumar M. (2019). Enhancement of the gut barrier integrity by a microbial metabolite through the Nrf2 pathway. Nat. Commun..

[168] Yang G., Bibi S., Du M., Suzuki T., Zhu M.J. (2017). Regulation of the intestinal tight junction by natural polyphenols: A mechanistic perspective. Crit. Rev. Food Sci. Nutr..

[169] Deaver J.A., Eum S.Y., Toborek M. (2018). Circadian Disruption Changes Gut Microbiome Taxa and Functional Gene Composition. Front. Microbiol..

[170] Henke M.T., Kenny D.J., Cassilly C.D., Vlamakis H., Xavier R.J., Clardy J. (2019). Ruminococcus gnavus, a member of the human gut microbiome associated with Crohn’s disease, produces an inflammatory polysaccharide. Proc. Nati. Acad. Sci. USA.

[171] Wang Z., Roberts A.B., Buffa J.A., Levison B.S., Zhu W., Org E., Gu X., Huang Y., Zamanian-Daryoush M., Culley M.K. (2015). Non-lethal Inhibition of Gut Microbial Trimethylamine Production for the Treatment of Atherosclerosis. Cell.

[172] Blackwood B.P., Yuan C.Y., Wood D.R., Nicolas J.D., Grothaus J.S., Hunter C.J. (2017). Probiotic Lactobacillus Species Strengthen Intestinal Barrier Function and Tight Junction Integrity in Experimental Necrotizing Enterocolitis. J. Probiotics Health.

[173] Chen L., Li H., Li J., Chen Y., Yang Y. (2019). Lactobacillus rhamnosus GG treatment improves intestinal permeability and modulates microbiota dysbiosis in an experimental model of sepsis. Int. J. Mol. Med..

[174] Krumbeck J.A., Rasmussen H.E., Hutkins R.W., Clarke J., Shawron K., Keshavarzian A., Walter J. (2018). Probiotic Bifidobacterium strains and galactooligosaccharides improve intestinal barrier function in obese adults but show no synergism when used together as synbiotics. Microbiome.

[175] Martin R., Laval L., Chain F., Miquel S., Natividad J., Cherbuy C., Sokol H., Verdu E.F., Vlieg J.v.H., Bermudez-Humaran L.G. (2016). Bifidobacterium animalis ssp lactis CNCM-I2494 Restores Gut Barrier Permeability in Chronically Low-Grade Inflamed Mice. Front. Microbiol..

[176] Miyauchi E., Morita H., Tanabe S. (2009). Lactobacillus rhamnosus alleviates intestinal barrier dysfunction in part by increasing expression of zonula occludens-1 and myosin light-chain kinase in vivo. J. Dairy Sci..

[177] Mujagic Z., de Vos P., Boekschoten M.V., Govers C., Pieters H.-J.H.M., de Wit N.J.W., Bron P.A., Masclee A.A.M., Troost F.J. (2017). The effects of Lactobacillus plantarum on small intestinal barrier function and mucosal gene transcription; a randomized double-blind placebo controlled trial. Sci. Rep..

[178] Stellwag E.J., Hylemon P.B. (1979). 7-ALPHA-DEHYDROXYLATION OF CHOLIC-ACID AND CHENODEOXYCHOLIC ACID BY CLOSTRIDIUM-LEPTUM. J. Lipid Res..

[179] Robben J., Parmentier G., Eyssen H. (1986). ISOLATION OF A RAT INTESTINAL CLOSTRIDIUM STRAIN PRODUCING 5-ALPHA AND AND 5-BETA-BILE SALT 3-ALPHA-SULFATASE ACTIVITY. Appl. Environ. Microbiol..

[180] Rau M., Rehman A., Dittrich M., Groen A.K., Hermanns H.M., Seyfried F., Beyersdorf N., Dandekar T., Rosenstiel P., Geier A. (2018). Fecal SCFAs and SCFA-producing bacteria in gut microbiome of human NAFLD as a putative link to systemic T-cell activation and advanced disease. United Eur. Gastroenterol. J..

[181] Selma M.V., Romo-Vaquero M., Garcia-Villalba R., Gonzalez-Sarrias A., Tomas-Barberan F.A., Espin J.C. (2016). The human gut microbial ecology associated with overweight and obesity determines ellagic acid metabolism. Food Funct..

